# Meaning in Life Among Patients With Chronic Pain and Suicidal Ideation: Mixed Methods Study

**DOI:** 10.2196/29365

**Published:** 2021-06-04

**Authors:** Alessandra Costanza, Vasileios Chytas, Valérie Piguet, Christophe Luthy, Viridiana Mazzola, Guido Bondolfi, Christine Cedraschi

**Affiliations:** 1 Department of Psychiatry Faculty of Medicine University of Geneva Geneva Switzerland; 2 Division of Clinical Pharmacology & Toxicology Multidisciplinary Pain Centre Geneva University Hospitals Geneva Switzerland; 3 Department of Psychiatry Service of Liaison Psychiatry and Crisis Intervention Geneva University Hospitals Geneva Switzerland; 4 Division of General Medical Rehabilitation Geneva University Hospitals Geneva Switzerland; 5 Department of Geriatrics and Rehabilitation Faculty of Medicine University of Geneva Geneva Switzerland

**Keywords:** suicide, suicidal behavior, suicidal ideation, suicide attempt, chronic pain, meaning in life, protective factors, risk factors, mental health

## Abstract

**Background:**

Patients with chronic pain have elevated risk of suicidal ideation and behavior, including suicide attempts and completed suicides. In most studies, associations between chronic pain and suicidal ideation/suicidal behavior are robust even after adjusting for the effect of sociodemographics and psychiatric comorbidity. However, to refine the risk profile of these patients, further exploration of other possible risk and protective factors is necessary.

**Objective:**

There is a common clinical observation that experiencing chronic pain often requires a revision of life goals and expectations, and hence, it impacts the existential domain including one’s perception of the meaning in life (MiL). This study aimed to characterize the main domains that constitute the personal MiL, including the “presence of” and “search for” constructs, in a group of patients with chronic pain and suicidal ideation.

**Methods:**

Seventy participants were enlisted by ongoing recruitment through a larger project anchored in daily clinical practice at the Multidisciplinary Pain Center of the Geneva University Hospitals. It was an observational mixed method study. Data were recorded through both validated quantitative questionnaires and qualitative open-ended questions.

**Results:**

The total sample consisted of 70 patients. Responses to questionnaires showed a depressive episode in 68 (97%) patients and anxious disorders in 25 (36%) patients. With a score threshold for positive MiL of 24, the mean score for the “presence of” construct was 20.13 (SD 8.23), and 63% (44/70) of respondents had a score <24. The mean score for the “search for” construct was lower at 18.14 (SD 8.64), and 70% (49/70) of respondents had a score <24. The “presence of” and “search for” constructs were significantly positively correlated (*R*=0.402; *P*=.001). An open question addressed the “presence of” construct by inviting the respondents to cite domains they consider as providing meaning in their life at the present time. All patients responded to this question, citing one or more domains. The three main dimensions that emerged from content analysis of this qualitative section were as follows: the domain of relationships, the domain of personal activities, and pain and its consequences on MiL.

**Conclusions:**

The study results provide insights into patients with chronic pain and suicidal ideation, including the domains that provide them with meaning in their lives and the impact of pain on these domains with regard to suicidal ideation. The main clinical implications concern both prevention and supportive/psychotherapeutic interventions. They are based on a narrative approach aiming to explore with the patients the content of their suffering and the MiL domains that they could identify to mitigate it, in order to restructure/reinforce these domains and thus possibly reduce suicidal ideation. Specifically, a focus on maintaining the domains of interpersonal relationships and personal activities can allow patients to ultimately escape the biopsychosocial vicious cycle of chronic pain–induced deep moral suffering.

## Introduction

Patients experiencing chronic pain are at elevated risk of suicidal ideation and behavior, including suicide attempts and completed suicides. It has been reported that there is a 20% to 40% prevalence rate of suicidal ideation, a lifetime prevalence between 5% and 14% of suicide attempts, and a double risk of death by suicide in patients with chronic pain as compared to controls [1-4].

In most studies, associations between chronic pain, suicidal ideation, and suicidal behavior have been demonstrated to be robust, even after adjusting for the effect of sociodemographics and psychiatric comorbidities, particularly depressive conditions [5-9]. A number of specific factors modulating the suicidal ideation and suicidal behavior risk in patients with chronic pain have been investigated (eg, pain characteristics, functional interference, illness beliefs, and access to opioids) [1,3,10]. However, the literature also highlights the need for further exploration of other possible risk and protective factors in order to refine the characterization of the risk profile in these patients [1]. In particular, in patients with chronic pain, protective factors have been less addressed than risk factors [11]. The interest in exploring protective factors in suicidal chronic pain patients arises from the common clinical observation that experiencing chronic pain often requires a revision of one’s life goals and expectations; hence, its impact affects the existential domain [12]. This perspective is of particular interest to provide clinicians the keys to identify relevant elements for the assessment of crisis factors and psychotherapeutic work, which can be important when making the decision to refer a patient to specialized care [13,14].

The historical intuitions of Frankl, who made his initial observations among prisoners of Nazi concentration camps, described the psychological construct of “meaning in life” (MiL) as a potent protective factor against suicide [15-17], with the individual having a “will of meaning” that presented a potent resiliency resource and the best chance to survive [15]. Afterwards, MiL was conceptualized under several theoretical perspectives, which pointed out different aspects of it. Among them, a recent model described MiL as “the web of connections, understandings, and interpretations that help us comprehend our experience and formulate plans directing our energies to the achievement of our desired future” [18]. This model divides MiL into two constructs, specifically “presence of” and “search for” MiL, which are not mutually exclusive [19]. However, somehow paradoxically, few studies have investigated MiL in relation to suicidal ideation and suicidal behavior [20]. Moreover, even fewer studies have addressed the contents of the subjective MiL in those individuals with suicidal ideation, that is, what makes or could have made their existence in some way meaningful [21,22]. Therefore, the aim of this study was to characterize the main domains that constitute the personal MiL in a group of patients with chronic pain and suicidal ideation.

## Methods

### Setting

This study was part of a larger research project investigating the role of demoralization and MiL in influencing suicidal ideation among patients affected by chronic pain [23]. This observational study was conducted by the Division of Clinical Pharmacology and Toxicology and the Service of Liaison Psychiatry and Crisis Intervention at the Multidisciplinary Pain Centre (MPC) of the Geneva University Hospitals. The MPC is a third-line ambulatory referral center where most patients are referred by their treating physicians for an interdisciplinary clinical evaluation and a review of the treatment proposals (eg, physical treatment, individual or group psychiatric/psychological treatment, and pharmacological proposals). Routinely, self-administered questionnaires were sent out to each participant before their first MPC visit, including the Beck Depression Inventory-II (BDI-II) [24]. Participants were enlisted by ongoing recruitment through a project anchored in daily clinical practice at the MPC.

The study included patients affected by chronic pain referred to the MPC and presenting with suicidal ideation. Patients were identified as having suicidal ideation by their positive response to question 9 of the BDI-II [24] that addresses suicidal thoughts or wishes with the following items: score 0, I don’t have any thoughts of killing myself; score 1, I have thoughts of killing myself, but I would not carry them out; score 2, I would like to kill myself; and score 3, I would kill myself if I had the chance. Patients whose scores ranged from 1 to 3 additionally underwent a clinical evaluation of suicidal ideation by a qualified team member (VC). This clinical interview determined the presence or absence of depression using a structured diagnostic interview to screen psychiatric diagnoses. The French version (5.0.0) of the Mini-International Neuropsychiatric Interview (MINI) [25] was used for DSM-IV disorders. Participants had to be ≥18 years old.

We excluded patients with an insufficient comprehension of the French language and those affected by dementia, psychotic disorders, or borderline personality as documented in the referral letter or as assessed during the first MPC visit.

### Statistical Analysis

During the first MPC visit, about 14 days after receiving the self-administered questionnaires at home, all patients who were identified by their positive response to question 9 of the BDI-II received a written communication informing them about the research project and a consent form. Patients were given at least 24 hours to review the written communication. At the subsequent study visit, a qualified team member (VC) further investigated the presence of suicidal ideation and responded to the patients’ questions about this study. Appropriate measures were taken for cases where severe suicidal ideation was identified using clinical evaluation and BDI-II, including accompanying the patient to the psychiatric emergency ward if necessary. All participants who provided written informed consent underwent face-to-face completion of the Meaning in Life Questionnaire (MLQ) [26]. Following the requirements of the observational study protocol [23], 70 patients with suicidal ideation were recruited.

### Instruments

The BDI-II and MLQ [26] were used. The MLQ is a self-reported multiple-choice inventory. The first section includes 10 items that measure the “presence of” construct of MiL (five items) and the “search for” construct of MiL (five items). Each item is rated on a 7-point Likert scale from “absolutely true” to “absolutely untrue,” leading to a score ranging from 5 (low MiL) to 35 (high) for each subscale. The MLQ does not have clear cut scores because it is intended to measure MiL across the complete range of human functioning. However, some probabilistic estimates about scores above or below 24 on the “presence of” and “search for” constructs have been provided on a website [27]. The MLQ has been translated into French and is available on this website. The closed section takes about 3 to 5 minutes to complete. The second section includes an open question asking the patient to cite three to seven domains that the patient considers as providing meaning to his or her life at the time being.

### Analysis

Descriptive statistical analyses were conducted to compute means and SDs for numerical variables and frequencies (%) for categorical variables. The chi-square test or Fisher exact test was used for categorical variables and checked for matching between groups regarding age, gender, and years of education. The post-hoc Tukey multiple-comparison test was used to find significant differences between means.

Patients’ responses to the open question of the MLQ were recorded and transcribed verbatim. Data analysis was performed by a medical doctor (psychiatrist) and a PhD psychologist (AC and CC) trained in qualitative procedures and with experience in qualitative studies. No prior relationships between data analysts and respondents were established before data collection.

The transcripts were analyzed using a manual data indexing technique to identify the key themes [28]. The qualitative content analysis began with individual close readings by the two researchers, who were working separately. The analysis continued throughout the coding process, using the constant comparative method, which consists of analyzing the responses by comparing one response with earlier observed ones [29]. This process was followed by comparisons of the readings, which were subsequently used to establish analytical categories and dimensions. A consensual process of reconciling differences in individual lists of categories led to an agreed set of categories [30]. These categories served as the basis for a grid that was then used to analyze the transcripts to maximize the theoretical sensitivity and rigor [31,32]. The sample of patients’ responses investigated allowed us to reach a point where no new categories emerged from the analysis. The categories were discussed and refined by consensus between the two researchers. Not unexpectedly, data saturation, defined as the point in data collection and analysis at which new information produces little or no change in the codebook [33], was reached. Data saturation was achieved after analyzing 40 responses. The next 30 responses confirmed data saturation and allowed further refinement of the grid. These categories and dimensions served as the basis for a final grid, which was used independently by the two researchers to analyze the transcripts. This assessment entailed an analysis of the data carried out by investigators with a different background (AC and CC) so that findings emerged from consensus between investigators. Interrater agreement was high (κ>0.92), and disagreements were solved by consensus. The matter at stake here may not be the degree of concordance between investigators but rather the insights that discussion can provide for refining coding frames [34].

In line with the recommendations for qualitative analysis reported in a language different from the one used for data collection, the interviews and data analysis were conducted in French, and the researchers used fluid descriptions of meanings in their discussions to achieve the best possible understanding of the concepts involved in the research [35].

### Ethical Considerations

The Ethics Committee of the Canton of Geneva approved the scientific utilization of collected data (project no. 2017-02138; decision dated January 25, 2018). The research project was carried out in accordance with the research plan and Swiss legal and regulatory requirements, in agreement with the principles stated in the current version of the Declaration of Helsinki and the Essentials of Good Clinical Practice issued by Public Health Switzerland. Written informed consent was obtained from all patients.

## Results

### Patient Sample Characteristics

Following the requirements of the observational study protocol, 70 patients were included in the study. Among the 70 participants, 42 were women and 28 were men, with a mean age of 54 years (Table 1). As the results of the BDI indicated, the participants experienced moderate to severe depressive symptoms (Table 1). The MINI validated the presence of a depressive episode in 68 out of the 70 patients, as well as the presence of anxious disorders in one-third of the group (25 out of 70 patients).

**Table 1:** Sociodemographic and clinical characteristics of the participants.

Characteristic		Value
Age (years), mean (SD; range)		54.3 (15.4; 20-85)
**Education, n (%)**		
	Compulsory school	13 (19%)
	Professional degree	27 (39%)
	High school	21 (30%)
	University	9 (13%)
**Employment status, n (%)**		
	Working (part time or full time)	10 (14%)
	Retired	6 (9%)
	Sick leave or state support	54 (77%)
Living alone, n (%)		26 (37%)
**Pain etiology, n (%)**		
	Neuropathic	44 (63%)
	Osteoarticular	22 (32%)
	Visceral or other	4 (6%)
Duration of pain (years), mean (SD)		8.06 (8)
**Pain intensity (visual analog scale score, range 0-10)**		
	At present	6.95
	At its best	5.33
	At its worst	8.91
Beck Depression Inventory (BDI) score, mean (SD)		31.3 (11.4)
**Extent of depressive symptoms (BDI), n (%)**		
	No depressive symptoms (<10)	2 (3%)
	Light (11-20)	10 (14%)
	Moderate (21-30)	24 (34%)
	Severe (>30)	34 (49%)

### “Presence of” and “Search for” MiL

As for the first part of the MLQ, the mean score for the “presence of” MiL was 20.13 (SD 8.23), and 63% (44/70) of the respondents had a score <24. The scores for the “search for” MiL were lower (mean score 18.14, SD 8.64), and 70% (49/70) of the respondents had a score <24. The “presence of” and “search for” MiL were significantly positively correlated (*R*=0.402; *P*=.001).

### Open Question Inviting Participants Linking MiL to Personal Domains

The second part of the MLQ addresses more specifically the “presence of” MiL as the open question invites the respondent to cite domains that he or she considers as providing meaning to his or her life at the time being, without referring to the constructs of “presence of” or “search for” MiL. It is noteworthy that all participants provided responses to the open question. The total number of responses was well over 70 as only about one-fifth of the patients mentioned ≤3 domains in their responses. Three main dimensions emerged from content analysis, that is, responses pertaining to (1) the domain of relationships, (2) the domain of activities, and (3) pain and its consequences on MiL.

#### Responses Linking MiL to Relationships

An overwhelming majority of the participants (n=65, 93%) mentioned relationships with the family, spouse, children (and/or grandchildren), and friends as a domain that provides meaning to their life. The idea of sharing (time, activities, and emotions) was in the foreground as in the following response:

My family and my friends. Their presence is very important for me, I feel considered… they give me love… Being valued in their eyes is important for me […]. Then the relationship with other people (outside of my family) also gives meaning to my life. Telling funny stories for example… having a good time with my family and friends or colleagues while sharing a meal…

respondent #29, 20-year-old man

Another domain was to be useful and thus be valued through the relationships, as the following responses show:

First, humanitarian work is very important for me and gives meaning to my life. Then also…contact with the loved ones I still have. I do not want to hurt them or disappoint them…

respondent #16, 69-year-old woman

My girlfriend who makes me think that I’m not alone… the wish to have children…help the others. I have always helped others, but my present situation does not allow me to do so… This gives me meaning but at the time being, I can no longer take care of myself, so I cannot help others…

respondent #63, 38-year-old man

Try to be happy and make people around me happy… try to give the best of myself to others… people call me ‘Mother Theresa’… sometimes I feel drained. My companion and my daughter also provide meaning to my life. I need to be useful for the others, to be able to help them be well or just be better. It is frustrating but I cannot do so because of my pain.

respondent #64, 58-year-old woman

These responses could be either embodied, that is, referring to one or more specified persons as in the examples above, or disembodied, that is, referring to relationships in general or as a general value or concept. An example response of the latter is as follows:

Having contacts with other people… sharing provides meaning to my life

respondent #4, 57-year-old man

Although the relationship may be something to hold on to, the interference of pain may be too overwhelming, as reflected in the following response:

My life has no particular meaning. My wife is the only element that helps me hold on a bit… I feel much more miserable when she is not there. Sadly, I do not talk enough with my wife… because I cannot…I suffer from being completely dependent on her. I try to spend time reading or watching TV, but that does not make sense to me…. I cannot enjoy it… I have no more pleasure… and I have no more hope to get better… I can no longer go out for a walk… I’ve become a bedridden sick old man.

respondent #20, 82-year-old man

#### Responses Linking MiL to Personal Activities

Personal activities were frequent responses in this group of patients given by about two-thirds of respondents in this category. These activities may serve various purposes, such as to feel better and engage in relationships. One patient made the following statement:

Try to improve my condition and do everything I can for that, go out, move, walk… gives meaning to my life… Also meet people, engage in activities with my daughter and friends.

respondent #12, 58-year-old woman

They may also represent a wish to change life and escape from pain and its consequences. The responses refer to travel, moving to a warmer place, moving somewhere closer to nature, and moving far from civilization. An example response is as follows:

Travel. I need to fly away…it is a kind of flight, a headlong rush.

respondent #54, 60-year-old woman

About one-quarter of respondents stressed that these activities have to be pleasant and enjoyable. An example response is as follows:

Playing sports that provide me with good sensations… it helps me balance everything that goes through my mind.

respondent #21, 67-year-old woman

However, activities may not be an appropriate response to giving meaning to one’s life, as one female respondent indicated, after having mentioned her family who provides her a feeling of belonging, and the energizing power of nature. The response was as follows:

There are other things that I enjoy such as reading, playing video games or taking pictures, but all this, it does not provide me with a meaning in life.

respondent #28, 60-year-old woman

Furthermore, more than one-quarter of respondents indicated that the identification of pleasurable and meaningful activities is often hampered by pain. An example response is as follows:

I think that I have understood the meaning of my life. It is my family, my friends and everything that gives me pleasure, eating, drinking, traveling, football… Yet, it is difficult to experience it because of my pain that is so intense.

respondent #11, 58-year-old man

#### Responses Linking MiL to Pain and Its Consequences on MiL

Indeed, as mentioned above, despite the presence of important and meaningful relationships and activities, the interference of pain may be overwhelming, to the point of erasing all other responses. One respondent made the following statement:

Nothing has meaning to me. What kind of meaning in life could I have? My pain does not let me enjoy anything for real…

respondent #32, 67-year-old man

About half of the respondents mentioned pain and its consequences explicitly. All of these consequences were described as deleterious, spoiling potential good moments and pleasure, and possibly leading to loss of objectives and MiL. An example response is as follows:

[…]…Getting better (without pain) and find a job and have a social life may offer me meaning, although I do no longer believe that these objectives can be reached… I am living one day after the other and I avoid making projects for the future. In fact, I am trying to give a meaning to my life, but sometimes I do not know exactly what I should search for…

respondent #5, 32-year-old woman

This lack or loss of objectives and meaning can have suicidal ideation as a counterpart as indicated by a patient:

There is nothing that really has sense in my life. I have no specific objective in life nor do I have any pleasure. My quality of life is zero. I keep up only thanks to my grandson because his mother is unable to care properly for him and I do not want to hurt him if I commit suicide… nothing can help me to get better…

respondent #6, 60-year-old woman

Finding MiL in spite of pain may be too difficult a task, as a patient responded:

My life has absolutely no meaning. I keep going only thanks to my family, but when pain is very intense, I do not think of them. I have no pleasure. My life is not worth living.

respondent #18, 46-year-old woman

Yet, when pain recedes, new perspectives seem to open up. An example response is as follows:

I have been feeling better since a few days, so meaning is back in my life… receive other people’s appreciation and respect… also various sources of pleasure in my life… and my family, but this is not as important as the other two domains…

respondent #13, 69-year-old man

The identification of these dimensions was independent of the respondents’ gender and age. Furthermore, there were no significant differences between respondents with neuropathic pain and those with osteoarticular pain. Since a substantial proportion of respondents presented with moderate or even severe symptoms of depression (58/70, 83%), the categories and dimensions mentioned above may be more representative of this particular group. Yet, it was mostly patients who were still working, either part time or full time, who mentioned “work” as an important activity. Patients who were still working also presented with lower scores in depression tests. Taken together, the results from this group of patients with chronic pain and suicidal ideation point toward shared rather than divergent views of what makes or could have made the respondents’ existence more meaningful.

## Discussion

### Principal Findings

To the best of our knowledge, this is the first exploration into MiL using a mixed-method approach characterized by the use of both a validated quantitative questionnaire and qualitative open-ended questions, in patients with chronic pain.

Scores for the first part of the questionnaire were low and did not reach the 24-point cutoff threshold for either the “presence of” or the “search for” MiL constructs [27]. These results are in line with the scores of the BDI that identified a high proportion of moderate to severe depressive symptoms in these patients, thus stressing the link between suicidal ideation and MiL in this group. Findings in the literature are controversial. “Presence of” MiL is rather uniformly thought to be beneficial [36]. By contrast, “search for” MiL appears less consensual, as some authors consider it the essence of human motivation [16] and others consider it a sign that one has lost meaning [37,38] or a feeling like one’s life has somewhat less meaning [19,26]. Recently, among patients with chronic pain, different MiL profiles have been characterized by combining various levels of “presence of” and “search for” MiL. Some patients were associated with a unique adjustment outcome. Those having profiles with high scores for “presence of” MiL showed fewer depressive symptoms and greater life satisfaction [37,38]. Both constructs have been found to be highly stable over time by the same authors, suggesting that MiL may reflect more a trait, rather than a state aspect, of individual functioning [38].

**Figure 1:**
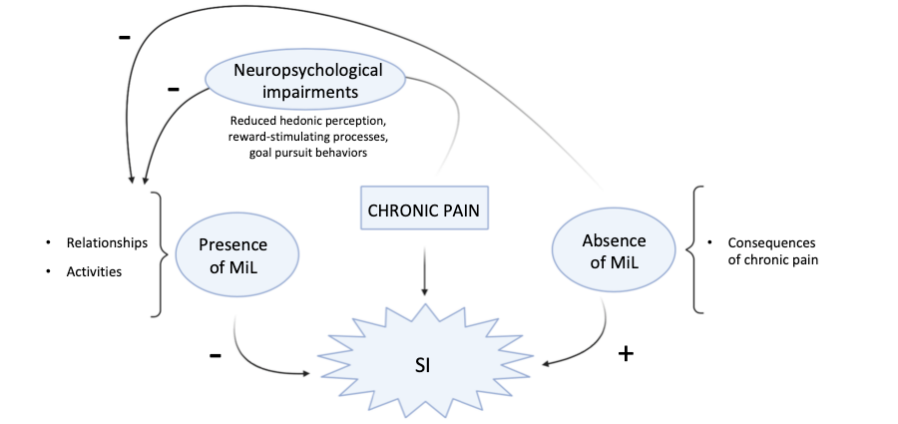
Pathways from chronic pain to suicidal ideation (SI): A vicious circle in a biopsychosocial framework. MiL: meaning in life.

When asked to mention domains that provide them MiL by open-ended questions, the typical responses of patients in our study were an illustration of the biopsychosocial model. Indeed, whether short or long, these responses addressed various dimensions. These dimensions included physical, emotional, and social aspects and were embedded in a timeframe where chronicity is central. In this regard, our results identified the following two critical dimensions that define MiL for patients with chronic pain: the presence of supportive interpersonal relationships and meaningful activities. When mentioned, pain was presented as having a major impact on the person’s daily life, possibly leading to mood disturbances, social isolation, decrease in activities, fear of the future, and a generalized sense of loss, as well as loss of both objectives and meaning. Pain may thus undermine MiL, directly or by actively limiting the seeking out for the two domains that confer MiL, in a vicious circle that leads to suicidal ideation (Figure 1).

Within this biopsychosocial framework, previous studies have documented the reduction of significant social relationships and personal activities among patients with chronic pain. A large-scale study of more than half a million participants in the United Kingdom showed that chronic pain could be reliably predicted by loneliness (odds ratio [OR] 1.843, 95% CI 1.816-1.870) and social exclusion (OR 2.314, 95% CI 2.249-2.380) [39]. Conversely, a longitudinal study of 1563 adults over the age of 60 years in the United States identified chronic pain as a diagnostic factor for the onset of loneliness as the odds for loneliness onset was 1.58 times higher among participants experiencing pain in comparison with pain-free participants, even after adjustment for other medical and psychosocial covariates [40]. Regarding activities, a community-based study in the United States of 591 patients with chronic pain stressed that this condition interferes significantly with productivity-related societal engagements as work performance in participants with chronic pain was markedly impaired [41]. Similarly, adults with chronic pain, either widespread or localized, showed a lower prevalence of engaging in leisure activities in comparison to those without chronic pain [42].

From a neurological perspective, chronic pain might interfere with the process of rewards, defined as matters that result in positive emotions and feelings. Laboratory studies have shown that acute pain can stimulate reward-related behaviors [43]. In patients with chronic pain, however, reduced hedonic perception in naturally rewarded behaviors has been documented [44]. Such a reduction in the perception of pleasure in patients with chronic pain might be attributed to reduced sensitivity to reward-stimulating processes [45].

Furthermore, goal pursuit behaviors, conceptualized as “internal representations of desired states, where states are broadly construed as outcomes, events, or processes” [46], could also be impacted by chronic pain. A study exploring the presence of daily goal conflicts in patients with fibromyalgia showed that these patients reported more pain-instigated disruption of goal-oriented daily activities (eg, household, social, and interpersonal goals) than healthy control individuals [47]. Collectively, these chronic pain-induced neuropsychological interferences might trigger a gradual loss of social interactions and activities.

The losses in these two domains are particularly salient in our study as patients clearly linked social interactions and activities to MiL, emphasizing that a loss in these domains could lead to a loss in MiL. This is of particular relevance as MiL has a protective role against the development of suicidal ideation and suicidal behavior. Following the pioneering studies of Frankl [15-17], recent studies have proposed this possible function of MiL among heterogeneous populations, including patients with chronic pain [23,48], patients attending a psychiatric emergency department (ED) [21,49,50], and community-dwelling individuals during the COVID-19 pandemic [51]. One of these studies [21] utilized the same qualitative approach as our study, but addressed patients attending a psychiatric ED. The findings of both studies converge. In both populations, relationships and activities were the main domains giving MiL to suicidal patients. These findings are consistent with findings from previous studies in the literature on the impacts of relationships and activities on reducing suicidal ideation and suicidal behavior.

The association between loneliness and suicidality is well documented. A recent extended review of the literature showed that both the objective condition (eg, living alone and social isolation) and the subjective feeling of being alone (ie, loneliness and alienation) were strongly associated with suicidal outcomes, such as suicidal ideation and suicidal behavior [52]. Notably, the subjective feeling had a major impact [52]. Two nationally representative samples (in the United States and in the United Kingdom) showed that social support was associated with a decreased risk of suicide (ORs of 0.7 and 0.9, respectively) [53]. In our study, a particular emphasis was placed on the presence of family members and, among them, of children/grandchildren and spouses. This is concordant with previous studies [21,52].

Our findings regarding the role of activities echo several prior studies suggesting that the lack of professional activities might also result in higher suicide risk [54]. In this regard, the interplay between professional activities and suicide is complex, involving some societal effects, such as the loss of social status, loss of connectedness, and lower income, all or some of which contribute to a higher vulnerability to suicide risk [55]. To the best of our knowledge, only one other study using qualitative data in the literature [21] has mentioned the relevance of other types of activities, although it is more related to personal interests and the intellectual/nonintellectual pleasure sphere, in comparison to our study.

### Limitations and Strengths

This study has several limitations. First, the sample size of this study was small, and future large-scale studies are required to substantiate the results regarding the closed-ended portion of the MLQ. However, the sample size allowed us to conduct a thorough analysis of the patients’ responses to the open-ended question investigating the domains that gave them MiL. Currently, we do not know how people with chronic pain but without suicidal ideation would identify the domains that give meaning to their lives. Yet, this study provides further information on the difficulties these patients face when trying to sort out what gives them MiL. Second, we did not analyze our findings in light of the participants’ heterogeneous sociodemographic characteristics. Therefore, it would be important to conduct further investigations to identify whether there are age- and gender-specific perspectives on MiL. We did find, however, that the characteristics of participants enlisted by ongoing recruitment through a project anchored in daily clinical practice at the MPC mirrored those of patients who arrived by referral. Indeed, the research was conducted in a third-line referral consultation. While our results may be transferable to patients having severe chronic pain associated with depressive symptoms, the transferability of our results to other clinical settings might still be difficult. However, the characteristics of our sample provide different perspectives on the issue at stake. Taken together, the results of this study have indicated several avenues that could be pursued by future investigations. For example, the sociodemographic and clinical characteristics of a sample could be used to refine the results presented here and to obtain a better understanding of the role of not only chronic pain and suicidal ideation, but also the various features of pain and its consequences.

It is noteworthy that this study made extensive use of investigator triangulation, with the final thematic analysis discussed within a multidisciplinary group (ie, psychiatry, psychology, clinical pharmacology, and internal medicine) so that findings were confirmed through consensus. Investigator triangulation allowed corroboration of the data, thus contributing to the credibility and validity of the findings [56]. The reliability of the findings was assessed by using patient-generated data via interviews and by verification of interpretation from a multidisciplinary group of researchers [57]. Concerning credibility, confirmability, and transferability of the results, research methods were derived from previous comparable projects, and familiarity with the culture and an adequate understanding of the participants were developed before the first data were collected. A further strength of this study was that its design allowed for an analysis based on two different sets of data investigating MiL, namely closed-ended questions and narrative responses. However, the open question does not specify whether it addresses the “presence of” or the “search for” construct, and this should be specifically investigated in a future study.

### Clinical Implications

There are various psychological approaches for chronic pain, but all have the same basic requirements as follows: patient adherence on the one hand and patient-therapist agreement on the definition of objectives that are both realistic and meaningful for the patient on the other hand. It is thus essential to involve the patient in this definition. This in turn implies that the therapist can express empathy and validate the patient’s pain and suffering.

In clinical interviews with patients with chronic pain, pain has to be thoroughly considered along with other aspects, such as activities that may require adaptations, as well as the possible fluctuations in the patient’s perception of MiL. As part of a negotiation process, the clinical interview also has to consider the patient’s resources and how he or she may try to cope with the situation with the clinician’s help, while still avoiding that the psychotherapeutic offer be perceived as a medical desertion. The results of our study stress the benefits of the MLQ as not only an indicator of psychological distress but also a tool that may be used in the patient-therapist discussion about the patient’s goals and sources of meaning. In this regard, such an instrument could help the clinician in the identification of relevant elements for the assessment of crisis factors that may be related to suicidal ideation. It may indeed contribute to the definition of common objectives and expected benefits for the treatment with respect to improvements in the patient’s quality of life and functional capacities.

### Conclusions

The results of this study provide the first insights into the views of patients with chronic pain regarding the domains that provide them with meaning in their lives and also into the impact of pain on these domains with regard to suicidal ideation. The main clinical implication concerns prevention and supportive/psychotherapeutic interventions, based on a narrative approach aiming to explore with the patients the contents of their suffering and the MiL domains that they could identify to mitigate them, in order to restructure or reinforce these domains [16,17]. Specifically, any treatment (also involving practical measures) focusing on maintaining the domains of interpersonal relationships and activities can possibly improve MiL and thus reduce suicidal ideation by allowing patients to ultimately escape this biopsychosocial vicious cycle of chronic pain–induced deep moral suffering.

## Abbreviations

BDI: Beck Depression Inventory
ED: emergency department
MiL: meaning in life
MINI: Mini-International Neuropsychiatric Interview
MLQ: Meaning in Life Questionnaire
MPC: Multidisciplinary Pain Centre
OR: odds ratio
